# Can OCT Angiography Be Made a Quantitative Blood Measurement Tool?

**DOI:** 10.3390/app7070687

**Published:** 2017-07-04

**Authors:** Jun Zhu, Conrad W. Merkle, Marcel T. Bernucci, Shau Poh Chong, Vivek J. Srinivasan

**Affiliations:** 1 Department of Biomedical Engineering, University of California Davis, Davis, CA 95616, USA; 2 Department of Ophthalmology and Vision Science, School of Medicine, University of California Davis, Sacramento, CA 95817, USA

**Keywords:** optical coherence tomography, angiography, scattering, red blood cells, rheology, imaging, hemodynamics, blood flow

## Abstract

Optical Coherence Tomography Angiography (OCTA) refers to a powerful class of OCT scanning protocols and algorithms that selectively enhance the imaging of blood vessel lumens, based mainly on the motion and scattering of red blood cells (RBCs). Though OCTA is widely used in clinical and basic science applications for visualization of perfused blood vessels, OCTA is still primarily a ***qualitative*** tool. However, more quantitative hemodynamic information would better delineate disease mechanisms, and potentially improve the sensitivity for detecting early stages of disease. Here, we take a broader view of OCTA in the context of microvascular hemodynamics and light scattering. Paying particular attention to the unique challenges presented by capillaries versus larger supplying and draining vessels, we critically assess opportunities and challenges in making OCTA a ***quantitative*** tool.

## 1. Introduction

The microcirculation comprises a network of blood vessels that delivers oxygen and nutrients to surrounding tissues, removes waste products and heat, and otherwise supports tissue viability [1–3]. Red blood cells (RBCs) are the main carriers of oxygen in blood. “Optical Coherence Tomography Angiography (OCTA)” is a term for the specialized Optical Coherence Tomography (OCT) scanning protocols and post-processing algorithms that mainly enhance the motion contrast of red blood cells (RBCs) in OCT images to selectively highlight these vessels. By enabling the visualization of cell-perfused vasculature without an exogenous contrast agent, OCT angiography has generated enormous interest in ophthalmology [4–11], gastroenterology [12,13], cancer biology [14,15], and neuroscience [16,17] over the past decade. It has been particularly useful in studying diseases where the microvascular morphology or presence of perfusion changes over time. However, with few exceptions [18–20], the majority of published studies have used OCT angiography *qualitatively*, primarily as a means of visualization. Here, we review the relevant basic hemodynamic principles, fundamentals of OCTA, categories of OCTA scanning protocols, and classes of OCTA algorithms. We argue that a rigorous and model-based relationship between hemodynamic parameters, light scattering theory, and measurement observables [21] in OCT angiography will pave the way towards more *quantitative* imaging of hemodynamics by OCTA and related methods, with the potential to enhance all applications.

## 2. OCTA Fundamentals

A unifying feature of all OCTA algorithms is that they visualize objects that are both moving and backscattering. Hence, we begin our review with a discussion of hemodynamics and light scattering properties of blood. Importantly, we distinguish between capillaries (<10 μm in diameter), where RBCs flow in a line and hematocrits are low, and macrovasculature, where RBCs flow side-by-side and hematocrits approach systemic levels, with the understanding that non-capillary microvessels (10–100 μm in diameter) represent an intermediate case between the two extremes discussed here [2,3].

### 2.1. Hemodynamic Parameters

**Figure 1:**
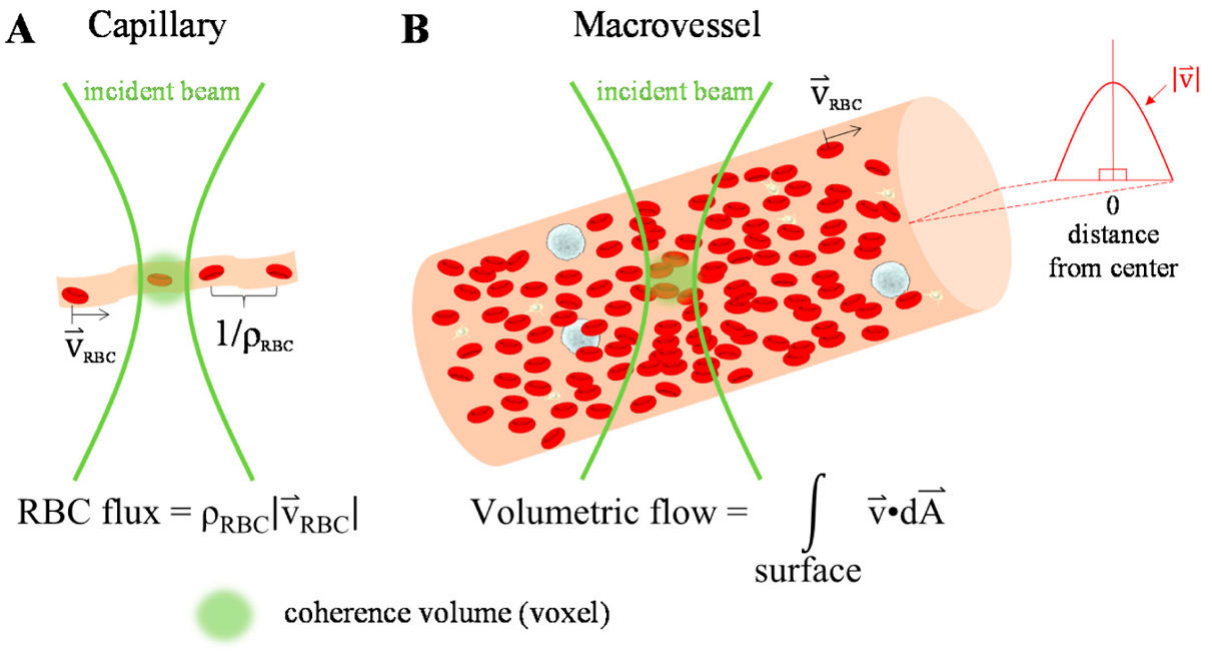
(**A**) Flow in capillaries (microvessels with diameters of <10 μm) is single-file, usually with highly variable hematocrits that fall below systemic levels; (**B**) On the other hand, macrovascular flow often follows a blunted laminar profile at near-systemic hematocrits. Consequently, different approaches are required to quantify microvascular versus macrovascular hemodynamics via OCTA (Optical Coherence Tomography Angiography) imaging.

What are the main hemodynamic parameters that impact observed OCTA signals? In capillaries (Figure 1A), the RBC flow is single-file, with plasma gaps in between [22,23]. RBC speed (distance/time), flux (#/time), and linear density (#/distance) are thus primary hemodynamic parameters in capillaries. Due to the plasma gaps between cells, flux can often be determined by imaging individual capillaries and counting RBCs traversing a single location [23]. Assuming single-file capillary flow, microvascular tube hematocrit (H_tube_), or RBC volume fraction, is related to linear density (ϱ) by ϱ = H_tube_A/V_RBC_, where V_RBC_ is the red blood cell volume and A is the vessel cross-sectional area. Capillary tube hematocrit is generally a factor of ~2–3*×* lower than systemic levels [24], but hematocrit can vary considerably between capillaries. In macrovessels (Figure 1B), which include supplying arteries and draining veins, the blood velocity varies across the vessel cross-section. In contrast to microvessels, macrovascular hematocrit approaches systemic levels of ~40–45% [25]. Flow is typically laminar with some degree of blunting [24], with the largest shear rate, or velocity gradient, at the edge of the vessel. In macrovessels, RBC velocity or speed (distance/time), flow rate (volume/time), and hematocrit (volume/volume) are the primary hemodynamic parameters. All hemodynamic parameters vary over time with respiration and the heartbeat of the subject [24].

### 2.2. Light Scattering from Red Blood Cells

**Figure 2:**
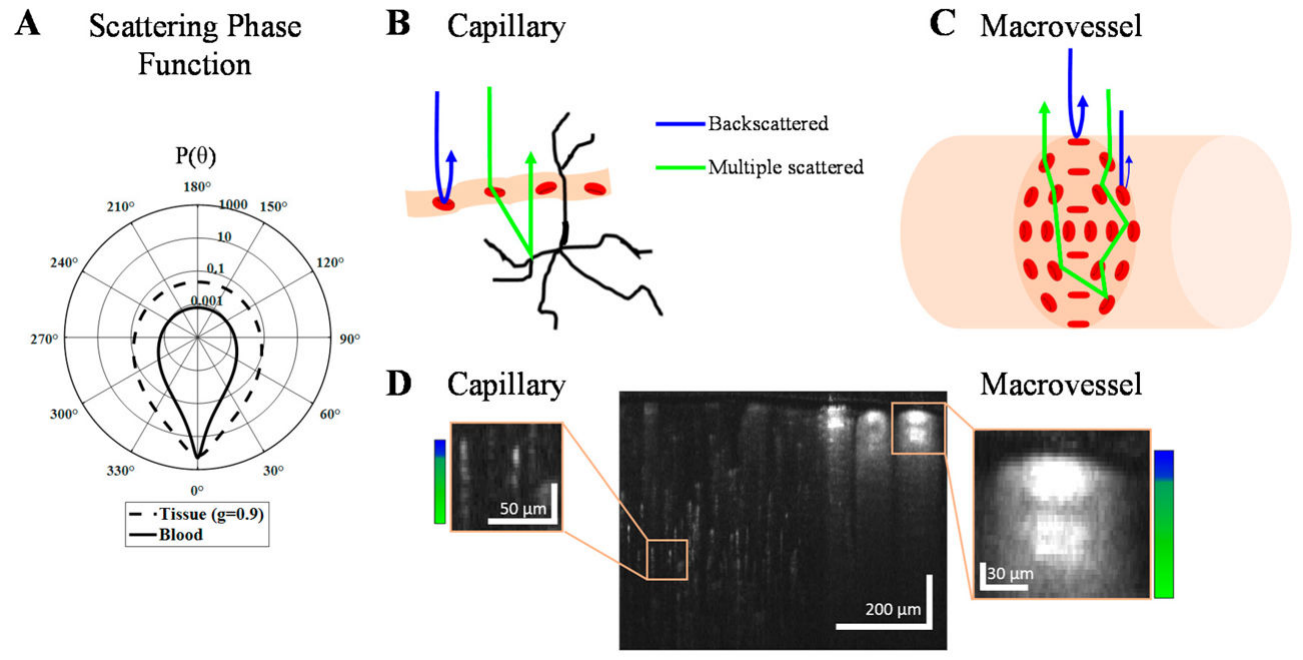
Single and multiple scattering in the OCTA of capillaries versus macrovasculature. (**A**) Blood has a high scattering anisotropy, leading to a high probability of detecting multiple scattered light paths; (**B**) For capillaries, dynamic RBC (red blood cell) forward scattering precedes or follows static tissue backscattering, which leads to “multiple scattering” tails; (**C**) In large vessels, the backscattering cross-section is determined by the shear-induced orientation of RBCs with their flat face parallel to the shear force. If the vessel lumen exceeds a scattering length, multiple intravascular dynamic scattering events (green) before detection are likely; (**D**) Cross-sectional OCT (Optical Coherence Tomography) angiogram of the mouse brain at 1300 nm (complex interframe subtraction method) with a qualitative colorbar showing the balance of backscattered light (blue) and multiple scattered light (green) in a capillary (left) and macrovessel (right).

What are the physical properties of RBCs that enable their detection by OCTA? RBC scattering and absorption properties derive from the presence of hemoglobin and its complex refractive index [26]. Major absorption bands of hemoglobin, related to the imaginary part of the complex refractive index, predominate at visible and shorter wavelengths, while hemoglobin absorption becomes negligible at near-infrared wavelengths, where scattering dominates. The light scattering properties of individual RBCs are determined by the refractive index contrast with respect to the surrounding plasma, as well as their shape and size relative to the medium wavelength. The real part of the complex refractive index, or refractive index, of hemoglobin is larger by ~3–6% relative to the surrounding plasma [26–28]. RBCs are biconcave disks (Figure 2), with a diameter of 6–8 μm and thickness of ~2 μm, although their shape changes under external stress. Due to the large volume fraction of RBCs and their refractive index mismatch relative to plasma, RBCs are the main scattering constituent in blood [27,29,30].

The scattering properties of both individual RBCs and ensembles of RBCs are important in OCTA. Due to their irregular shape, the probability of light scattering in a given direction for a particular RBC depends on both its orientation and the direction of incident light. An ensemble of RBCs with different orientations can be characterized by a scattering coefficient (μ_s_), the scattering probability per unit distance; a scattering phase function (P(θ)), the probability of scattering in a given elevation direction θ per unit solid angle; and a scattering anisotropy (g = E[cos(θ)]), the expectation or average (E[ ]) of cos(θ) over solid angle. These parameters characterize scattering of whole blood, which comprises an ensemble volume of RBCs with random orientations. In whole blood, empirically measured g and μ_s_ include dependent and multiple scattering effects [31]. With a hematocrit of around 45%, whole blood is found to be highly forward scattering between 750 and 950 nm, with a scattering coefficient (μ_s_) between 65 and 80 mm^−1^, and anisotropy (g) between 0.97 and 0.99 [29,32–34]. Exemplary phase functions [30,32,35] for tissue (Henyey-Greenstein with g = 0.9) and blood (Gegenbauer-Kernel with g = 0.972 and α = 0.49 [36]) are shown in Figure 2A on a logarithmic scale. Tissue has a higher probability of back scattering than blood, while blood is considerably more forward scattering.

In OCTA (Figure 2B,C), detected light ideally results from paths with single RBC backscattering (θ = 180°) events (blue). However, the high RBC anisotropy (Figure 2A) makes detection of multiple scattered light (green) likely. Probable light paths can be understood through the principles of radiative transport. In capillaries, where RBC flow is single-file, light forward scattered from RBCs is also backscattered from extravascular tissue (Figure 2B), creating axial multiple scattering tails (Figure 2D left box). In macrovessels, there are two important effects. First, RBCs tend to align their flat face parallel to the shear force, i.e., facing outwards along the vessel circumference (Figure 2C). The largest backscattering cross-section occurs when the shortest RBC dimension is aligned with the incident light. Therefore, the signal is enhanced at the top and bottom of the vessel lumen and reduced at the side (Figure 2D right box) [37]. At higher shear rates, RBCs elongate and the backscattering pattern disappears [38]. Second, for vessel lumens larger than a scattering length (1/μ_s_), multiple intravascular dynamic scattering events (green) can occur before detection.

As OCTA images are created by post-processing OCT data, OCTA has an image penetration depth comparable to or less than OCT. This is typically ~0.5–1.5 mm in most tissues, depending on the source wavelength and the sample optical properties [39,40]. It is important to note that while OCTA visualizes blood vessels, the penetration depth of OCTA may be determined by the attenuation of both intravascular and extravascular tissue.

## 3. OCTA Signal

In this section, we provide a unifying framework for the OCT signal to facilitate the discussion of OCTA algorithms in Section 4. Commonly-used symbols or variables and their definitions are summarized in Table 1, while other symbols are defined in the text.

All standard OCTA algorithms [41,42] start from the complex OCT signal. The complex, depth-resolved OCT signal can be expressed as:

(1) S(x,z,t)=∣S(x,z,t)∣exp{i∅(x,z,t)}.

Note that S(x, z, t) is related to the depth-resolved optical field, integrated over a resolution element (coherence volume). Therefore, the depth-resolved intensity, I(x, z, t), is equivalent to the magnitude square of the field, i.e., I(x, z, t) = *|*S(x, z, t)*|*^2^. OCTA algorithms may operate on either S(x, z, t), ∅(x, z, t), or I(x, z, t) as the “signal”, and accordingly, can be categorized into complex field-based techniques, phase-based techniques, and intensity-based techniques. In its simplest form, OCTA employs differences between OCT signals at the same spatial position over a series of time points to highlight scatterer motion. As discussed in Section 2.2, RBCs are the main blood scattering component. Due to the dynamic motion of RBCs, the overall field, phase, and intensity fluctuate. For the field, these variations are determined, in a statistical sense, by the first-order field autocorrelation function, r(τ), in which r(τ) = R(τ)/R(0) and R(τ) = E[S(x, z, t + τ)S * (x, z, t)], where E[ ] represents expectation and τ is the time lag. Under some circumstances, all other signal variations, including those of the intensity and phase, derive their statistical properties from the field autocorrelation [43].

**Figure 3:**
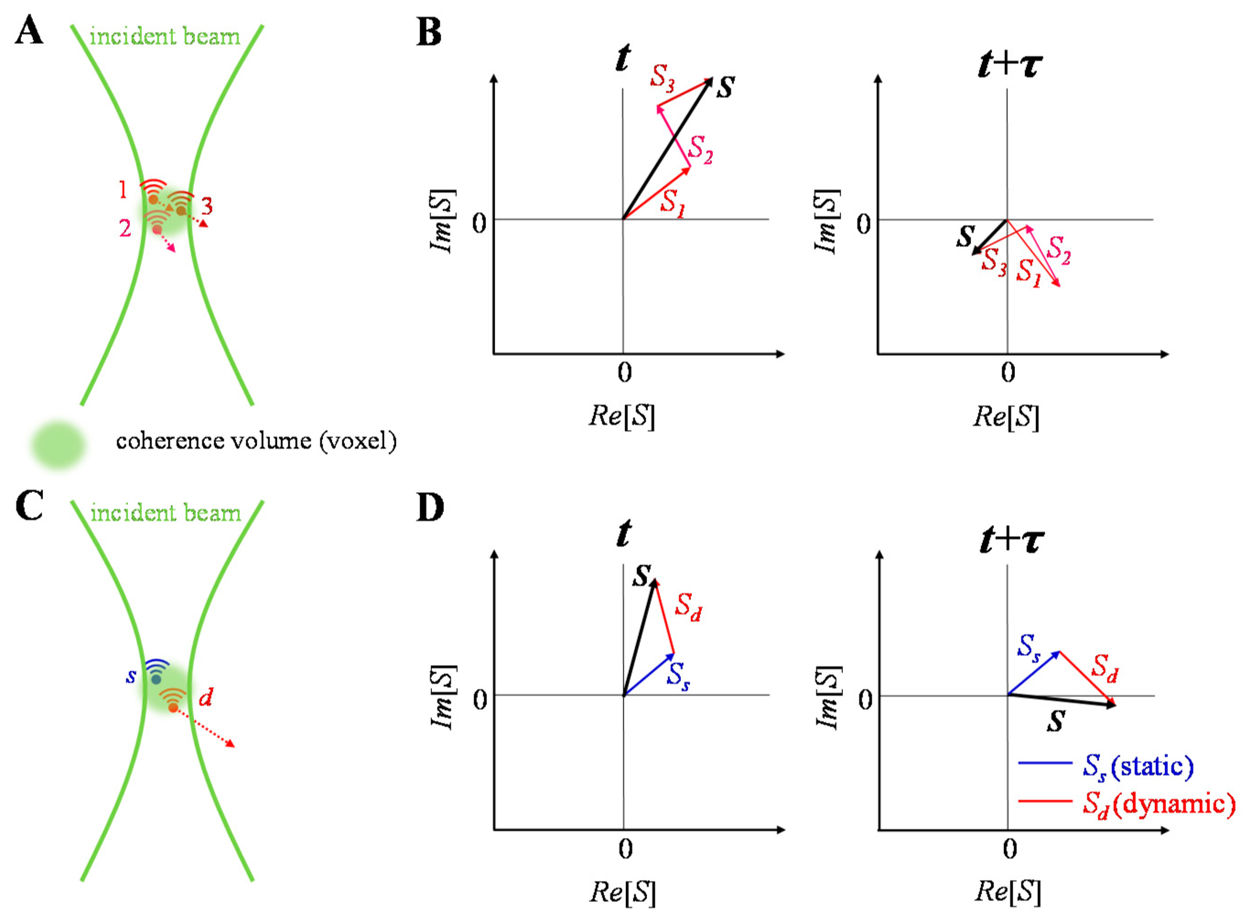
The motion of scatterers in a coherence volume gives rise to complex field fluctuations that form the basis for OCTA signals. The contributions to the complex field are shown at two different points in time (t and t + τ). (**A**,**B**) Field fluctuations due to dynamic scatterers in a coherence volume. (**C**,**D**) Field fluctuations due to a combination of static (blue) and dynamic (red) scatterers in a coherence volume.

OCT complex signal dynamics are illustrated in Figure 3. The complex signal is treated as a complex summation of backscattered fields from individual scatterers within the coherence volume. The coherence volume is defined by the beam waist in the transverse direction and the coherence length in the axial direction. Changes in the fields from individual scatterers over time leads to changes in the total signal over time (Figure 3A,B). In many practical situations, scatterers may be further classified as “dynamic” and “static” depending on whether they move or not, with both scatterer types contributing to the signal in the same coherence volume (Figure 3C,D).

**Figure 4:**
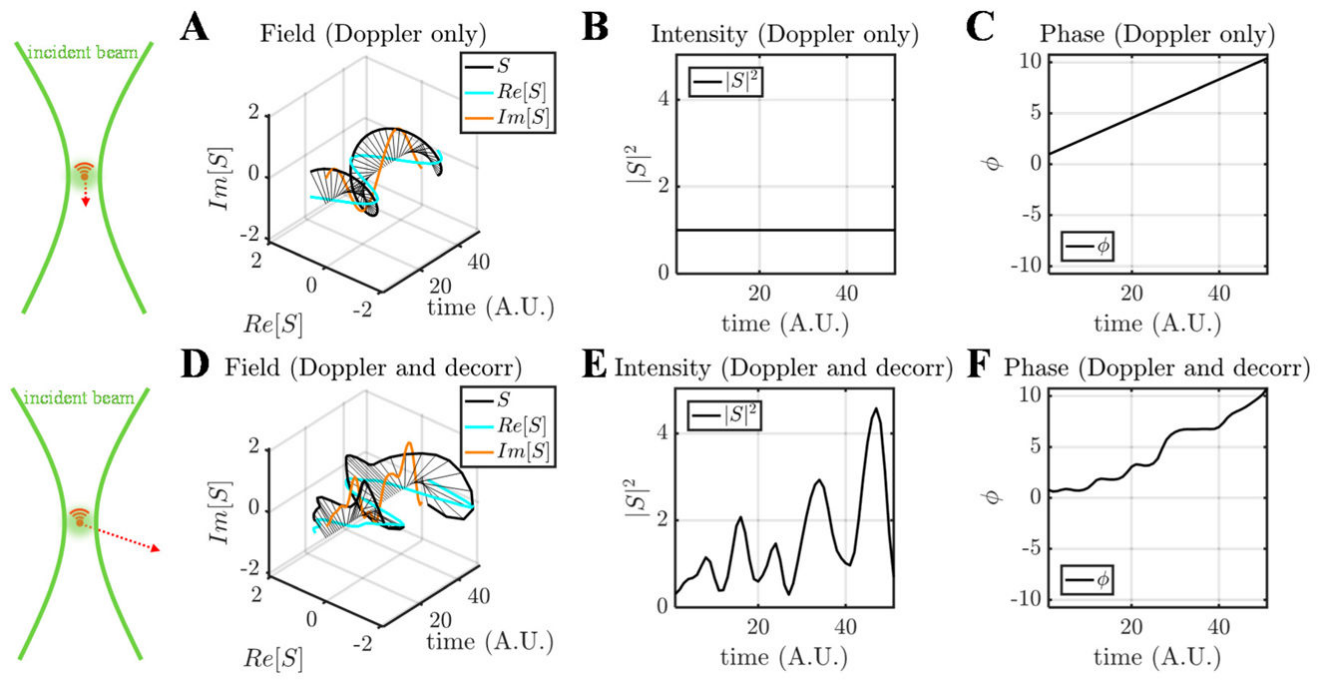
The major categories of OCT signal fluctuations are Doppler shifts and decorrelation. Comparison of complex field, intensity, and phase time courses, for the case of a pure Doppler shift (**A**–**C**) and a Doppler shift with decorrelation (**D**–**F**). For a pure Doppler shift, the field traces out a helical pattern (**A**), whereas decorrelation introduces random deviations from this pattern (**D**).

The nature of scatterer dynamics plays a major role in determining the OCT signal changes (Figure 4). Generally, scatterer motion is accompanied by both a Doppler shift and decorrelation [44]. When the scatterer has an axial velocity component, moving towards or away from the incident beam, the complex field rotates, tracing a helix over time (Figure 4A–C). This effect can be described as a linear phase shift over time due to the Doppler effect, or a “Doppler phase shift”. When the scatterer is undergoing a dynamic conformational change, rotation, or translational motion through the coherence volume, both the OCT signal intensity and phase change randomly (Figure 4D–F). This random change of the complex field is known as decorrelation. Doppler shifts are associated with a change in the phase of the complex field autocorrelation, while decorrelation is associated with a decrease in the magnitude of the complex field autocorrelation, *|*R(τ)*|*, with increasing τ.

**Figure 5:**
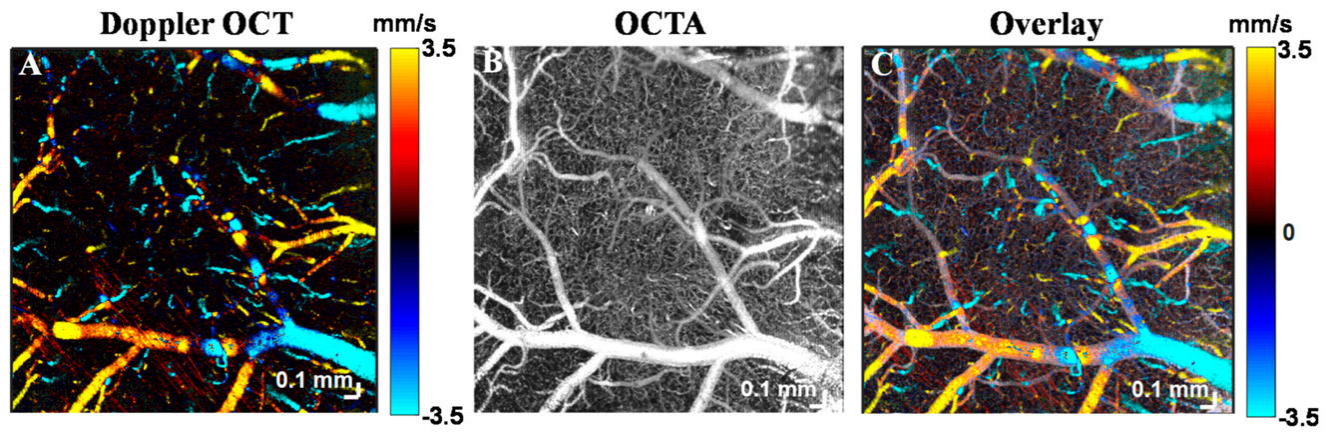
Doppler OCT and OCTA in the mouse brain. (**A**) Doppler OCT can visualize flow based on Doppler shifts, caused by motion in the axial direction, towards or away from the probe beam. On the other hand, OCTA visualizes flow based on decorrelation, usually caused by translational motion through the coherence volume, as well as Doppler shifts. The overlay of both methods (**C**) shows that Doppler OCT is mainly limited to ascending venules or descending arterioles, where Doppler shifts dominate. On the other hand, OCTA, which is sensitive to decorrelation, more comprehensively shows vasculature, including regions with predominantly transverse flow. A standard Kasai algorithm was used on transversally oversampled images for (**A**) and a complex interframe subtraction method was used on rapidly acquired repeated cross-sectional images for (**B**).

Both Doppler shifts and decorrelation are present to varying degrees in all vasculature. Note that a Doppler shift due to translational axial motion through the coherence volume implies decorrelation. On the other hand, decorrelation occurs even for transverse motion or rotation, and does not necessarily imply a Doppler shift. To illustrate this, Figure 5 shows a comparison between Doppler OCT and OCTA of mouse brain microvasculature. Doppler OCT detects phase changes caused by translational axial motion [45]. The requirement for axial phase shifts renders Doppler OCT only sensitive to motion parallel to the incident beam. Doppler shifts predominate in larger microvessels which are ascending or descending (Figure 5A); hence when used for angiography, the Doppler effect provides only a partial microvascular map. By comparison, decorrelation involves random deviations of the complex field and predominates in vessels with transverse flow. Thus, OCTA, which senses decorrelation via intensity and/or phase, more comprehensively shows the vasculature (Figure 5B,C).

**Figure 6:**
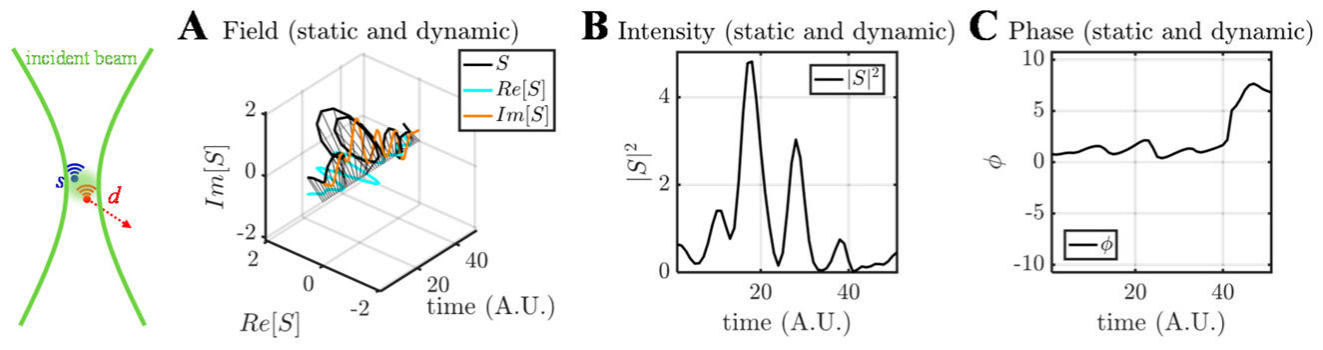
Comparison of complex field (**A**), intensity (**B**), and phase (**C**) time courses when both static and dynamic scattering are present in a coherence volume (with Doppler shift and decorrelation of the dynamic component). Such coherence volumes are present at the edges of vessels.

Finally, it should be noted that the presence of static scattering can significantly alter time courses. The OCT field, intensity, and phase time courses due to dynamic scattering in the presence of a static scatterer are shown in Figure 6. As suggested by Figure 3D, the presence of static scattering confines the field fluctuations to a portion of the complex plane (Figure 6A). As will be discussed in Section 7.1, the possible presence of static scatterer(s) must be considered in order to recover quantitative information about the Doppler phase shift or the decorrelation rate in OCTA.

## 4. OCTA Algorithms

The previous section showed that OCTA signals depend on the type of dynamics (Doppler shift or decorrelation), the observed parameter (intensity, phase, or field), and the possible presence of static scattering in the coherence volume. With this discussion in mind, we now present the main classes of angiography algorithms.

### 4.1. Intensity- or Amplitude-Based OCTA Algorithms

Intensity-based OCTA algorithms use I(x, z, t) = *|*S(x, z, t)*|*^2^, while amplitude-based OCTA algorithms use *|*S(x, z, t)*|* in Equation (1).

The first class of intensity-based OCTA algorithms is the speckle variance method. Speckle [46] can be described as the random interference of scattering fields (indexed by m) that cannot be resolved within a coherence volume:

(2) $$S(z)=\sum_{m}S_{m}(z),$$

S_m_ represents the fields within a coherence volume, each weighted according to the point spread function at the scatterer location (Figure 3). The intensity (as well as the phase and field) changes over time as the configuration of scatterers changes, causing decorrelation (Figure 4D–F). Decorrelation can occur as RBCs pass through a coherence volume, but may also occur due to rotational motion or diffusion. In 2005, Barton and Stromski showed the feasibility of flow speed measurement without phase information by evaluating speckle pattern changes [47]. In 2008, Mariampillai et al. [48] used interframe speckle variance to visualize microcirculation. In [48], speckle variance was defined as:

(3) $$\text{SV}(x,z)=\frac{1}{N}\sum_{t=0}^{(N-1)T}{\left[I(x,z,t)-\bar{I(x,z)}\right]}^{2},$$

where t = 0, T, 2T, . . . , (N − 1)T represents the OCT acquisition time; T is the time interval; N is the total number of acquisitions at the same position; x and z denote lateral and depth indices, respectively; and $\bar{I(x,z)}=\bar{{|S(x,z)|}^{2}}$ is the time-averaged intensity at position (x, z). This is a temporally averaged, variance-based algorithm without normalization. By ignoring the phase in Equation (1), the method is not sensitive to pure Doppler shifts. Consequently, speckle variance is not susceptible to phase noise. However, the speckle variance method may be compromised due to interframe bulk tissue motion. While in-plane (xz) motion can be compensated in principle, out-of-plane motion is more challenging to correct. To minimize motion effects, later in 2010, Mariampillai et al. [49] optimized the frame number and frame rate for a given level of bulk tissue motion, through maximizing the speckle variance signal-to-noise ratio (SNR) between a “dynamic” and “static” pixel. Speckle variance SNR is calculated as:

(4) $${\text{SV}}_{\text{SNR}}(N,\bar{I(x,z)})=\frac{{\text{SV}}_{\text{dynamic}}(N,\bar{I(x,z)})-{\text{SV}}_{\text{static}}(N,\bar{I(x,z)})}{\sqrt{\sigma_{\text{dynamic}}^{2}(N,\bar{I(x,z)})+\sigma_{\text{static}}^{2}(N,\bar{I(x,z)})}},$$

where $\bar{I(x,z)}$ is the time-averaged intensity for both “dynamic” and “static” pixels, SV_dynamic_ and SV_static_ are speckle variances calculated from Equation (3), and $\sigma_{\text{dynamic}}^{2}$ and $\sigma_{\text{static}}^{2}$ are variances of SV_dynamic_ and SV_static_, respectively. By optimizing the frame number under conditions of low tissue bulk motion, capillaries can be reliably detected [49].

As the dynamic tissue signal has a lower temporal correlation, at a given time lag, than static tissue, correlation has been investigated as a parameter for angiography. In 2011, Enfield et al. [50] demonstrated in vivo human volar forearm imaging of the capillary density and vessel diameter with correlation mapping optical coherence tomography (cmOCT). The correlation between OCT frames acquired at time t and t + T at the same position is:

(5) $$\text{cmOCT}(x,z)=\sum_{p=0}^{V}\sum_{q=0}^{W}\frac{\left[I(x+p,z+q,t)-\bar{I(t)}][I(x+p,z+q,t+T)-\bar{I(t+T)}\right]}{\sqrt{{\left[I(x+p,z+q,t)-\bar{I(t)}\right]}^{2}+{\left[I(x+p,z+q,t+T)-\bar{I(t+T)}\right]}^{2}}},$$

where V and W define the extent of the spatial region for correlation calculation, and $\bar{I(t)}$ denotes the spatially averaged intensity over this region. This is a spatially averaged, correlation-based algorithm with normalization. After this calculation, a 2D correlation map can be formed by applying a threshold to binarize the image into static and dynamic regions. In 2012, Jia et al. [51] proposed split-spectrum amplitude-decorrelation angiography (SSADA) to image the human macula and optic nerve head. Ensuring a nearly isotropic coherence volume size by splitting the spectrum to degrade the axial resolution to equal the transverse resolution, they then applied a method similar to cmOCT.

### 4.2. Phase-Based OCTA Algorithms

Phase-based OCTA algorithms rely on ∅(x, z, t) in Equation (1) to distinguish dynamic and static tissue. Doppler OCT, a category of phase-based OCTA, uses a deterministic Doppler phase shift for in vivo blood flow measurements [52,53]. While Doppler OCT can quantify flow, visualization applications are limited due to its angle dependence (Figure 5A). For instance, retinal blood vessels are nearly perpendicular to the optic axis, particularly outside of the optic nerve head, yielding insufficient phase shifts for Doppler measurements [42]. Power Doppler [54,55] and phase variance imaging [56] represent alternative approaches that are sensitive to decorrelation, or random non-deterministic Doppler shifts. In 2007, Fingler et al. [57] proposed phase variance for motion contrast. In [58], the phase variance at position (x, z) is defined as:

(6) $$\text{PV}(x,z)=\frac{1}{N-1}\sum_{t=0}^{(N-2)T}{\left[\Delta \emptyset (x,z,t)-\bar{\Delta \emptyset (x,z)}\right]}^{2}.$$

The phase difference at a given location is given by:

(7) Δ∅(x,z,t)=∅(x,z,t+T)-∅(x,z,t),

where T is the time lag. Equation (6) is a temporally averaged, variance-based algorithm without normalization. Phase-based OCTA algorithms are advantageous over amplitude- and intensity-based algorithms if phase changes but intensity and amplitude do not. However, phase-based OCTA loses information about the OCT signal amplitude and intensity. Moreover, phase-based OCTA may not detect changes in the presence of a large static scattering component. Similar to amplitude- and intensity-based algorithms, phase-based OCTA is sensitive to decorrelation (Figure 4E,F). However, as phase is particularly sensitive to axial motion, additional bulk motion phase correction is typically required. In [57], before phase variance analysis, Fingler et al. removed the bulk motion phase change:

(8) $$\Delta \emptyset^{\text{corr}}(x,z,t)=\Delta \emptyset (x,z,t)-\Delta \emptyset^{\text{bulk}}(x,t),$$

where Δ∅^corr^(x, z, t) denotes the corrected phase change, and Δ∅^bulk^(x, t) represents the phase change due to bulk motion, estimated as:

(9) $$\Delta \emptyset^{\text{bulk}}(x,t)=\frac{\sum_{z=a}^{b}\left[|S(x,z,t)|\Delta \emptyset (x,z,t)\right]}{\sum_{z=a}^{b}\left[|S(x,z,t)|\right]}$$

The phase change due to bulk motion is thus calculated by a weighted mean from z = a to z = b in one A-scan. Note that bulk phase change estimation based on cross-correlation is also possible [16,59].

### 4.3. Complex Signal-Based OCTA Algorithms

Complex signal-based OCTA algorithms use S(x, z, t), the complex field, which includes both the intensity/amplitude and phase in Equation (1). As both intensity and phase fluctuations (Figure 4B,C,E,F) arise from field fluctuations (Figure 4A,D), we assert that the complex field is more fundamental than either the intensity or phase. In particular, the static component can be readily handled in the complex domain (Figure 6). Also, unlike intensity-based OCTA, complex signal-based OCTA is sensitive to slow flow with only phase changes [60]. In 2007,Wang et al. [61] demonstrated complex signal-based OCT angiography, also called optical microangiography (OMAG), for the first time, while interframe complex OCTA was introduced later [16,62]. The most basic complex OCTA algorithm is based on subtraction,

(10) ΔS(x,z,t)=∣S(x,z,t+T)-S(x,z,t)∣,

where S(x, z, t+T) and S(x, z, t) are complex OCT signals acquired at the same position separated by a time lag T. This is a difference-based algorithm without normalization. Spatial or temporal averaging may be applied as needed. This expression may also be generalized as a variance calculation (or high-pass filter [16]) that eliminates static scattering:

(11) $$\Delta S(x,z)=\frac{1}{N}\sum_{t=0}^{(N-1)T}{|S(x,z,t)-\bar{S(x,z)}|}^{2}.$$

This is a temporally averaged, variance-based algorithm without normalization. As static and dynamic scatterer fields add in the complex domain (Figure 6), the above expression correctly eliminates static scattering to quantify the dynamic scattering signal.

**Figure 7:**
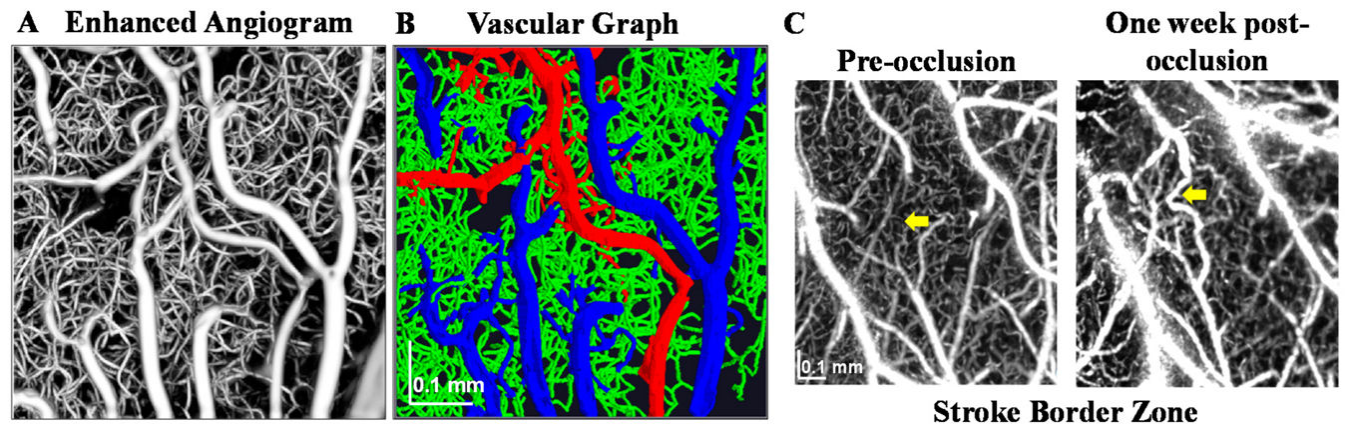
OCTA microscopy of the mouse brain enables an assessment of vascular connectivity (**A**,**B**) and longitudinal monitoring of microvascular remodeling (**C**) one week after distal middle cerebral artery occlusion (yellow arrow).

**Figure 8:**
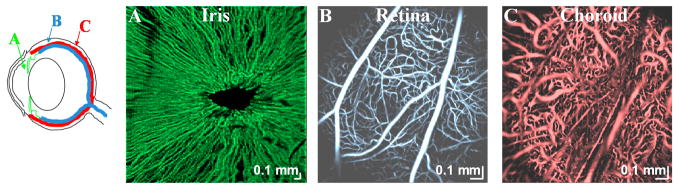
Ocular OCTA of iris (**A**), retina (**B**), and choroid (**C**). Hessian vesselness enhancement was applied to retinal and choroidal vasculature before display. Note that the pupil was dilated prior to OCTA acquisition for (**B**,**C**).

**Figure 9:**
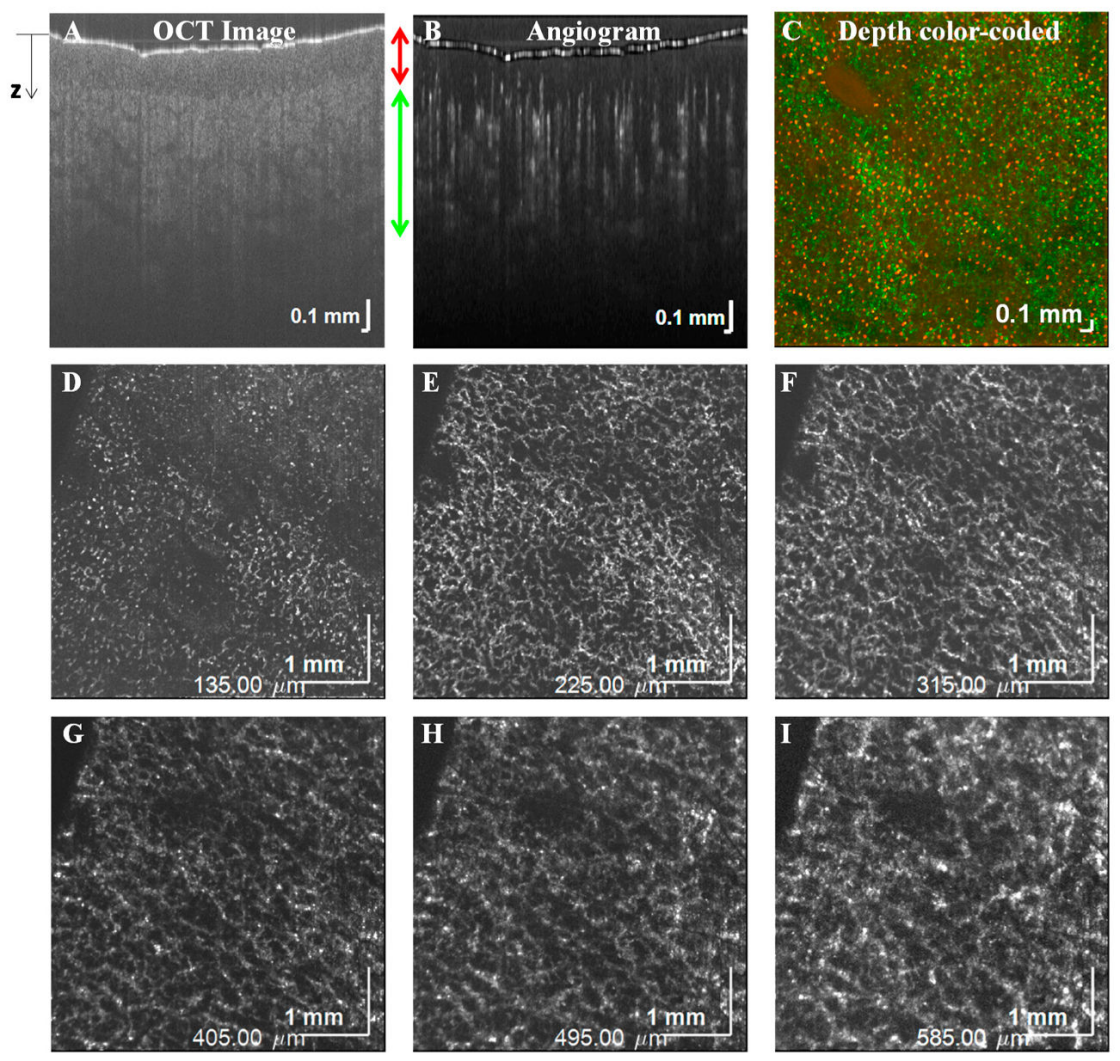
OCTA of the skin on a pig ear. OCT cross-sectional intensity image (**A**) and angiogram (**B**) determined by complex subtraction. (**C**) Overlay of superficial vessels in the epidermis (red) with deeper vasculature in the dermis (green). (**D**–**I**) Maximum intensity projections centered at different axial (z) positions relative to the surface.

Using a complex signal-based algorithm, several applications of OCTA are demonstrated here. Figure 7A,B shows OCTA graphing of the mouse brain vasculature in vivo. Longitudinal monitoring of recovery in the mouse brain, one week after an experimental ischemic stroke, is shown in Figure 7C. Note the presence of vascular remodeling (yellow arrows). Figure 8 shows OCTA of a rodent eye in vivo. Figure 9 presents OCTA of pig ear skin, including a cross-sectional intensity image (Figure 9A), cross-sectional angiogram image (Figure 9B), color-coded angiogram of superficial and deep vasculature (Figure 9C), and angiograms centered at different depths (Figure 9D–I). All figures employ a complex interframe subtraction algorithm for angiography.

In 2014, Nam et al. [63] proposed a complex differential variance (CDV) algorithm. This differential variance algorithm, applied to the OCT signal at a position (x, z), is:

(12) $$\text{CDV}(x,z)=\sqrt{1-\frac{\sum_{t=0}^{(N-2)T}|\sum_{k=-L}^{L}w_{k}S(x,z-k,t)S^{*}(x,z-k,t+T)|}{\sum_{t=0}^{(N-2)T}\sum_{k=-L}^{L}w_{k}\frac{1}{2}[I(x,z-k,t)+I(x,z-k,t+T)]}},$$

where w_k_ is a depth-dependent window function of length 2L + 1. Though it is referred to as a “variance” method, this algorithm is actually a spatially and temporally averaged, correlation-based method with normalization (see the discussion of variance versus correlation in Section 4.4). The correlation is estimated by averaging on a complex basis axially (in z) and a magnitude basis over time. Also note that the correlation definition is the complex conjugate of that used elsewhere in this paper, though due to the absolute value operation, this minor discrepancy has no effect on the final CDV.

### 4.4. Classification of Present OCTA Algorithms

Historically, all of the OCTA algorithms described above were novel at the time they were introduced. However, with the benefit of hindsight, we propose basic categories to classify OCTA algorithms in Table 2.

The primary distinction between algorithms, discussed in Section 4.1, Section 4.2, Section 4.3, is the OCT signal(s) employed. The second distinction, which is emphasized in the literature, is between variance/difference-based methods and correlation-based methods. However, here we argue that in some cases, this distinction is meaningless. Difference-based methods are actually estimating the following:

(13) $$D(T)=E[{|X_{t+T}-X_{t}|}^{2}],$$

where D(T) denotes the difference at a time lag of T and X_t_ can be the OCT field, intensity, or amplitude at time t. Variance-based methods are estimating the following:

(14) $$V=E[{|X_{t}-E(X_{t})|}^{2}].$$

On the other hand, the un-normalized autocorrelation is defined as:

(15) $$R(T)=E[X_{t}*X_{t+T}].$$

Further expanding Equation (13), the difference can be written in terms of the autocorrelation:

(16) $$D(T)=E[{|X_{t+T}|}^{2}]+E[{|X_{t}|}^{2}]-2\text{Re}\{E[X_{t}*X_{t+T}]\}=2R(0)-2\text{Re}\{R(T)\}.$$

Therefore, difference and correlation methods are very closely connected if R(T) is real. If R(T) is complex, as would be the case if X_t_ represented the field and Doppler shifting were present, the difference D(T) depends only on the real part of R(T). From Equations (14) and (15), it can be readily shown that R(0) = V if E[X_t_] = 0. Thus, every difference method corresponds to an equivalent correlation method via Equation (16).

The third distinction between algorithms is the way that the expectation, E[ ], is realized in practice. One method of realizing the expectation is by averaging over time. Another way is by averaging over space, at different tissue locations. Yet another way is spectral or optical wavelength averaging, employed in split-spectrum methods [51]. Under the assumption of ergodicity [64], all averaging methods are asymptotically equivalent, and in practice, all can be used to some degree. However, note that averaging over one dimension will automatically degrade the resolution in that dimension.

Fourth, OCTA methods can be distinguished by the use of normalization. The normalized correlation is divided by the signal power, R(0):

(17) $$r(T)=R(T)/E[{|X_{t}|}^{2}]=R(T)/R(0)$$

For the complex signal, the power R(0) is related to the total scattering within a coherence volume. In a vessel, this depends on the backscattering cross-section of RBCs (which depends on orientation according to Section 2.2), and the RBC density (hematocrit). As discussed further in Section 7.1, *|*R(τ)*|* is a monotonically decreasing function under certain conditions, with the decorrelation rate, or rate of autocorrelation decay, being proportional to speed. As difference methods depend on R(0) and R(T), there are two regimes to consider in understanding Equation (16). The first is when T is much longer than the intrinsic decorrelation time. In this case, R(T) ≪ R(0), and D(T) is proportional to the signal power R(0), typically related to backscattering (RBC density and orientation). If T is on the order of the intrinsic decorrelation time, the difference D(T) depends on both the signal power R(0) and the decorrelation rate. In this case, the interpretation of the difference D(T) becomes more ambiguous, and it can be affected by the signal power or decorrelation rate, which can be impacted by the RBC density, orientation, and speed. With the normalization in Equation (17), r(T) is more directly related to the rate of decorrelation, and hence, the RBC speed. However, to rigorously account for the possible presence of static scattering, measurements at several time lags [18] are required.

## 5. OCTA Scanning Protocols

**Figure 10:**
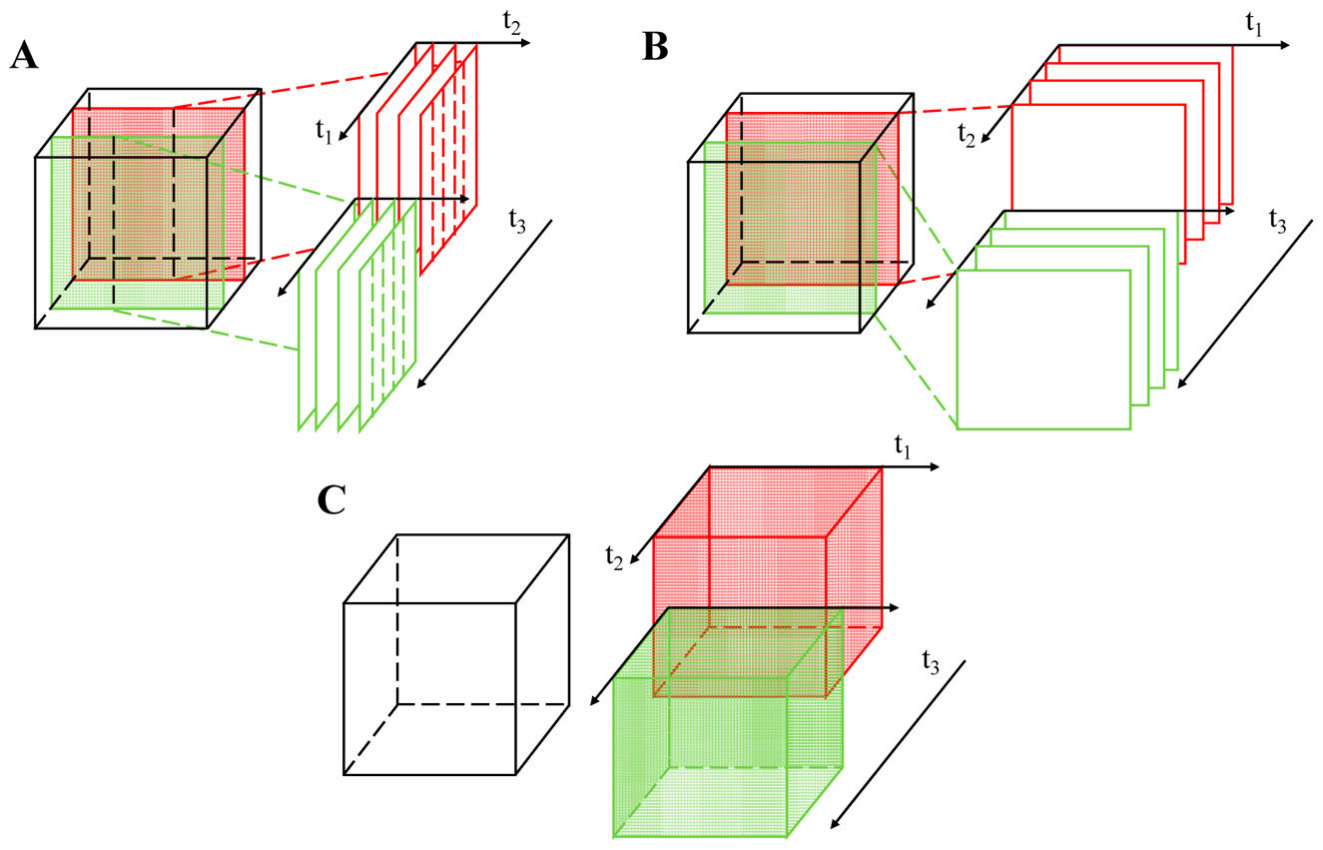
Volumetric OCTA scanning protocols can operate with respect to A-scan (**A**), frame (**B**), or volume (**C**). A cubic volume is scanned with time scales t_1_, t_2_, and t_3_. Data acquired sequentially along time scale t_3_ are shown in red and green. (**A**) MB-scan: multiple A-scans are obtained at one lateral position before switching to the next lateral position; (**B**) BM-scan or interframe scan: multiple B-scans are obtained at one cross-sectional location before switching to the next location; (**C**) intervolume scan: successive scans of the whole volume. Each scan achieves a progressively larger observation time for a single spatial position (t_3_ > t_2_ > t_1_).

The efficiency and sensitivity of OCTA measurements are determined by the OCTA scanning protocol. At a fundamental level, scanning protocols can be categorized based on whether the analysis is performed on consecutive A-scans, frames, or volumes. Figure 10 shows the so-called MB-scan, BM-scan and intervolume scanning methods. In Figure 10, the cube represents the imaged object, and t_1_, t_2_, t_3_ are the first, second, and third OCT scanning time scales, respectively, with t_3_ > t_2_ > t_1_. Each protocol can be characterized by the time duration for which a single location is observed.

To our knowledge, Fingler et al. [57] were the first to rigorously compare different OCTA scanning patterns. They compared the MB-scan (Figure 10A) and the BM-scan (Figure 10B), using a phase contrast algorithm. An M-scan is a repeated zero-dimensional scan at a single position, while a B-scan is a one-dimensional scan along a single axis. An MB-scan comprises multiple A-scans taken at one lateral position before switching to the next position (Figure 10A), while a BM-scan comprises repetitive B-scans taken along the same cross-section (Figure 10B). According to [57], the advantages and disadvantages of the two scanning methods are described here.

The MB-scan is an extension of Doppler OCT protocols. By increasing N, the number of A-scans per M-scan, the dynamic range for the measurement increases. However, the MB-scan is not time-efficient, because the total observation time for a single location is ~t_1_. Unless the dwell time is very long, *|*r(t_1_)*|* ~ 1; thus it is challenging to observe decorrelation. However, due to the rapid repeated sampling of the same position, the MB-scan can sample fast Doppler velocities [57] without aliasing.

On the contrary, a BM-scan compares consecutive frames, thereby more efficiently utilizing the total acquisition time. With a BM-scan, the total observation time for a single location is ~t_2_. In [57], the BM-scan was able to acquire data 200 times faster than an MB-scan of the same size. Even when using fast systems, the BM-scan may suffer from aliasing of fast Doppler velocities; however, the decorrelation rate can be obtained if the interframe time is short enough, i.e., *|*r(t_1_)*|* > 0, and provided that t_2_ exceeds the intrinsic decorrelation time.

In a logical extension of the above two scanning methods, in 2016, Wei et al. [65] proposed a volumetric optical microangiography method (Figure 10C) which used intervolume OCT scans to extract dynamic changes. The total observation time for a single location is ~t_3_. However, in this volumetric protocol, all information about the decorrelation rate is lost as speckles decorrelate between volumes (i.e., *|*r(t_2_)*|* ~ 0) for all but the slowest flows. Nevertheless, the volumetric OCTA is likely to become more prevalent as imaging speeds continue to improve [66].

## 6. Empirical Validation of OCTA

A major question in quantitative OCTA is the degree to which the measured signals are affected by the RBC speed versus density or orientation. Several authors have attempted to answer this question empirically. In 2016, Choi et al. [67] investigated the relationship between OMAG (complex difference OCTA) signals and capillary flow. They proposed an analytic model that expressed OMAG signals as a function of time interval between successive B-scan frames, particle speed, and concentration (the last two determine flux). Based on this model, they performed simulations, as well as phantom experiments, using microfluidic channels filled with diluted Intralipid solution to model blood vessels. It was shown that OMAG signal increases with flow speed within a certain range that depends on the time interval between successive B-scan frames, as expected based on Equation (16). Furthermore, OMAG signal increased with particle concentration, but was not strictly linear. One limitation of this study is that the Intralipid solution and blood possess very different scattering properties [68,69]. Su et al. [70] used blood samples in microfluidic channels to demonstrate the relationship between SSADA decorrelation signal and the flow speed and channel width. They concluded that before saturation, the decorrelation rate was proportional to the blood flow speed when the channel width was fixed.

Even if flow velocities, channel widths, and particle/cell concentrations are realistic, controlled ex vivo experiments are limited in how well they can model the range of phenomena that are present in vivo. These include effects such as static scattering and multiple scattering involving extravascular tissue (Figure 2B), RBC orientation and transit deformation, vascular compliance, and cell-endothelium interactions. So, in vitro experiments may verify algorithms under model conditions, but the model might only partially capture the range of rheological and hemodynamic phenomena present in vivo.

One proposed in vivo benchmark for OCTA is fluorescence angiography (FA), which is a gold standard method for perfusion imaging [7,42]. Comparative OCTA-FA studies [7,55] have suggested that the presence of moving blood cells is a prerequisite for detection by OCTA. The threshold red blood cell density and speed required for OCTA detection are usually determined by the algorithm sensitivity. While FA shows plasma perfusion, limited depth resolution and lack of three-dimensional data and quantitative flow information make FA a less-than-ideal technique for OCTA validation.

The gold standard for single vessel hemodynamic imaging in deep tissue is multiphoton microscopy (MPM) [71,72]. In the simplest implementation, a fluorescent label is injected into the bloodstream and volumetric two-photon microscopy (TPM) is performed to acquire an angiogram. Vakoc et al. [15] showed that OCTA and two-photon microscopy angiogram morphologies correlate well for vessels larger than capillaries, and that OCTA is not confounded by dye leakage, which can impair TPM. Aside from morphology, TPM line scans enable red blood cell imaging in individual capillaries [22], measuring in vivo speed, flux, and linear density quantitatively. In 2012, Srinivasan et al. [18] performed OCTA and TPM line scans sequentially in the same vessels in vivo, showing that OCTA decorrelation rate increases with RBC speed measured by TPM. Later in 2014, Wang et al. [73] validated OMAG (complex difference OCTA) with TPM, finding no significant difference between the respective vessel densities derived from OMAG and TPM, up to the penetration depth of TPM.

When comparing OCTA and TPM, it is important to recognize that their contrast mechanisms are complementary. As OCTA measures RBC scattering and TPM measures plasma tracer fluorescence, measurements of vessel diameter must disagree in small vessels due to the plasma only, cell-free layer [2]. Moreover, typically, OCTA has a worse volumetric resolution than TPM, and asynchrony in measurements [18] can additionally confound comparisons between modalities unless physiology is carefully maintained. Thus, rigorous verification of OCTA with simultaneous TPM is a promising topic for further investigation.

The most appealing and direct validation approach is to use another OCT modality or algorithm to cross-validate OCTA. In 2012, Ren et al. [74] noticed that the passage of a red blood cell through the OCT coherence volume led to phase and intensity transients. Based on this insight, they developed a particle counting method for measuring the flux, speed, and linear density in a capillary. Using particle counting, they developed and validated a phase intensity mapping (PIM) algorithm for measuring quantitative cerebral blood flow (CBF) [75]. It remains unclear whether individual red blood cell passage can be measured at all locations in an image, or whether these results are merely anecdotal. Moreover, the intensity pattern created by decorrelation can create random transients that could be easily mistaken for RBC passage (e.g., Figures 4E and 6B). Still, particle counting remains an attractive approach for validating OCTA in stable preparations.

## 7. OCTA Measurements of Hemodynamics

Based on dynamic changes in intensity, phase, or complex signal, OCTA algorithms can distinguish dynamic tissue from static tissue. Thus, while OCTA can answer the question “where is there flow?”, it cannot yet reliably answer the question “how much flow is there?”. In recent years, several attempts have been made to further quantify OCTA signals. Many of these efforts are based on estimating the autocorrelation function. While the autocorrelation function can be estimated, to date, there is no rigorous theory or model for recovering RBC flow or speed from OCTA signals. Here, we summarize some promising work towards these goals.

### 7.1. Flow Quantification

In 2010,Wang et al. [19] made an early effort at providing an autocorrelation model to measure transverse particle flow speed. Though they focused on intensity transients, here we generalize their initial work. The basic principle of their model is that when particles pass through the imaging beam, they create OCT signal transients that may provide information about the speed of the underlying particles. However, with a large coherence volume, the individual transients may overlap in time. The complex signal at position (x, z) is expressed as a superposition of particle contributions:

(18) $$S(x,z,t)=\sum_{k=1}^{G(x,z)}M_{k}(x,z)\text{REC}(x,z,t-t_{k}),$$

(19) $$\text{REC}(x,z,t)=\left\{ \begin{array}{cc} 1, & 0\leq t\leq \tau_{0}(x,z)\\ 0, & \text{otherwise} \end{array}\right.,$$

where k is index of the kth particle, G(x, z) is the total number of particles passing through the imaging beam within the signal acquisition period, M_k_(x, z) is the complex amplitude of the kth particle transient, t_k_ denotes the time when a particle begins to pass through the beam, and τ_0_(x, z) is the position-dependent transit time of the particle.

After expressing the complex OCT signal in terms of particle contributions, the normalized autocorrelation function of S(x, z, t) is given by:

(20) $$\frac{R(x,z,\tau )}{R(x,z,0)}=\left\{ \begin{array}{c} 1-\frac{\tau }{\tau_{0}(x,z)},\tau \leq \tau_{0}(x,z)\\ 0,\;\tau \geq \tau_{0}(x,z) \end{array}\right.,$$

where R(x, z, τ) is the autocorrelation function of S(x, z, t) with time lag τ. Note that this is equivalent to the normalized autocorrelation of REC(t). The slope of the normalized autocorrelation function in Equation (20) is proportional to the transverse speed (~1/τ_0_). Note that Equation (20) can be further generalized to accommodate other transient shapes.

In 2012, Srinivasan et al. [18] proposed an alternative model to relate the autocorrelation to speed. For small particles undergoing isotropic motion through a coherence volume, they proposed that the autocorrelation decay is determined by axial and transverse point spread functions, while for large particles, the spatial characteristics of the particles themselves dominate the autocorrelation as described above. In [76], for small particles, the autocorrelation function at time lag τ in cylindrical coordinates (ϱ, φ, z) is:

(21) $$R_{d}(\tau )=\frac{2{|K|}^{2}}{\pi^{2}w_{\varrho }^{4}}\sqrt{\frac{\pi }{(\frac{v_{\varrho }^{2}}{w_{\varrho }^{2}})+\frac{v_{z}^{2}}{w_{z}^{2}}}}P_{A}\exp\;\left[-\frac{{\left(v_{\varrho }\tau \right)}^{2}}{w_{\varrho }^{2}}-\frac{{\left(v_{z}\tau \right)}^{2}}{w_{z}^{2}}\right]\exp[i(\frac{4\pi n}{\lambda_{0}})v_{z}\tau ],$$

where w_ϱ_ is the transverse beam profile, w_z_ is the axial resolution, K is an arbitrary complex constant [77], P_A_ is the power in the random process which describes the field, v is the particle’s speed, n denotes the refractive index, and λ_0_ is the central wavelength. The power spectral density, P_d_, derived from the temporal autocorrelation function, is expressed as:

(22) $$P_{d}(f)=\frac{2{|K|}^{2}}{\pi w_{\varrho }^{4}(\frac{v_{\varrho }^{2}}{w_{\varrho }^{2}}+\frac{v_{z}^{2}}{w_{z}^{2}})}P_{A}\exp[-\frac{\pi^{2}{\left(f-\frac{2{\text{nv}}_{z}}{\lambda_{0}}\right)}^{2}}{\left(\frac{v_{\varrho }^{2}}{w_{\varrho }^{2}}+\frac{v_{z}^{2}}{w_{z}^{2}}\right)}].$$

In the presence of static scattering (Figure 6), the autocorrelation takes the form:

(23) $$R(\tau )=R_{d}(\tau )+R_{s}(\tau ),$$

where R_s_(τ) is the autocorrelation of the static component, with a much longer decorrelation time than the autocorrelation of the dynamic component, R_d_(τ). In practice, R_s_(τ) is usually constant over time scales of interest. Aside from the Doppler shift, the un-normalized autocorrelation R_d_(τ) provides two essential observables: the decorrelation rate, which is sensitive to speed, and power (P_A_), which is sensitive to the RBC density. Recent work has proposed to quantify OCTA using difference algorithms measured at several time delays [20,78], providing the ability to measure blood flow speed. Since difference and correlation algorithms are related by Equation (16), these algorithms essentially estimate the un-normalized autocorrelation. As highlighted in Equation (23), static scattering, if present, must also be taken into account in parametric estimations based on the autocorrelation.

Finally, a major limitation of existing models is that they do not account for multiple scattering. In particular, multiple dynamic scattering events (Figure 2C, green) increase the decorrelation rate relative to the single scattering models described above, as each dynamic scattering event causes momentum transfer [79]. In such cases, the decorrelation rate depends on the number of scattering events, which in turn is impacted by the RBC density. Thus, with multiple intravascular scattering events, decorrelation rate is not a “pure” metric of speed. Therefore, decorrelation rate is not a good metric of speed within macrovessels where multiple scattering dominates, but may perform better in capillaries where hematocrits are lower and singly backscattered light prevails (Figure 2B).

### 7.2. Hematocrit Quantification

Since OCTA signal depends on the RBC density, can OCTA be used to quantify hematocrit? The differences in rheology, geometry, and light scattering in capillaries versus macrovessels suggest different approaches for each. In macrovessels, backscattering or attenuation (signal slope) are possible observables which may help to determine hematocrit. However, due to the high scattering coefficient and anisotropy of RBCs, multiple scattering events are very likely, except at superficial path lengths (Figure 2C,D). In particular, at physiological hematocrits, dependent scattering and shadowing effects lead to a highly nonlinear relationship between the RBC concentration and scattering coefficient [80,81]. This nonlinear relationship hampers efforts at quantifying hematocrit based on light scattering and the signal slope alone. Additionally, the oxygen saturation dependence of hemoglobin refractive index and RBC scattering further complicate efforts to measure hematocrit based on attenuation [26]. The orientation-dependence of light scattering from RBCs (Figure 2C) makes quantifying hematocrit from backscattering alone challenging. Thus, quantification is challenging in macrovessels.

The single file flow and relatively lower hematocrit in capillaries makes multiple scattering within these vessels less problematic. However, RBCs may re-orient themselves and possibly deform as they squeeze through the smallest diameter capillaries, thereby changing their backscattering cross-sections. Moreover, measuring backscattering directly would need absolute calibration, which can be difficult in vivo. However, backscattering may still measure relative changes in the red blood cell content in capillaries [82] and, possibly, at the surfaces of macrovessels over time. Thus, while quantification of hematocrit changes is possible in capillaries, absolute measurements of hematocrit with conventional OCTA are currently challenging.

## 8. Can OCTA Be Made a Quantitative Tool?

OCTA systems can observe dynamic signal power (variance) and decorrelation rate [19,83], based on the dynamics of light scattering. As algorithms, imaging system performance, and motion tracking/compensation continue to improve, OCTA observables, particularly decorrelation rate, can be precisely and accurately measured. These observables may generate useful diagnostic information, even if their underlying hemodynamic correlates remain unclear. However, if OCTA observables can be directly linked to hemodynamic parameters such as blood flow, volume, hematocrit, and speed, OCTA diagnostics could aid understanding of pathogenesis. This effort requires an appropriate model to describe OCTA signals. The model may be empirical (Section 6), but ideally, should have a theoretical foundation (Section 7.1). Current theoretical models are very simple, and only account for single scattering [18,19,83,84]. Improvements in OCTA theory to include multiple scattering [85] and orientation effects [37] are needed. Empirical models have been developed for flow phantoms [67], but they may be limited to in vitro conditions, and their applicability in vivo remains uncertain. Better in vivo validation experiments, perhaps in well-controlled and stable animal preparations, are needed. Last, due to differences in light scattering and hemodynamics (Figures 1 and 2), models for capillaries and macrovessels must be developed independently.

In spite of these proposed efforts, the inherent complexity of the rheology and light transport in microvasculature may prevent reliable quantification of OCTA. Therefore, we propose that alternative optical properties (aside from light scattering) may enable more quantitative OCTA. For instance, visible light OCTA [86] enables direct absorption-based measurements of hemoglobin concentration, which is expected to correlate well with hematocrit (RBC volume fraction) under most conditions [87]. Yet another way to circumvent the pitfalls of RBC scattering is to introduce an exogenous contrast agent with more desirable scattering properties into the bloodstream [88]. If a more isotropically scattering contrast agent such as Intralipid^®^ [89,90] is used, angiograms derived from the contrast agent signal alone do not suffer from multiple scattering tails [89,90]. Microbubbles [91,92] are promising for enhancing intravascular scattering signals, and may present more well-defined decorrelation characteristics than blood. Moreover, if the contrast agent behaves like plasma and the signal can be calibrated and related to concentration [89,90], plasma flow, transit time, and volume can all be measured.

## 9. Conclusions

Despite recent strides in OCTA imaging speed, field-of-view, and measurement of OCTA observables, OCTA remains a qualitative tool at present. The obstacles to quantification include the irregular shape of RBCs, the consequent orientation-dependence of RBC backscattering, and the high anisotropy of the RBC scattering phase function, which leads to multiple scattering in large vessels. Quantification of OCTA signals can be achieved only through a rigorous understanding of the relationship between hemodynamics, rheology, and light scattering of RBCs. Improvements in theoretical models, validated in microvasculature in vivo against gold standard techniques and possibly in simulation, may help to improve this understanding. Finally, alternative measurements, based on absorption or exogenous contrast agents, may help to alleviate some of the confounds associated with RBC scattering and enhance the quantitative information provided by OCTA. More quantitative interpretation of OCTA would aid the application of this promising technique to study pathophysiology, and also potentially enhance the clinical impact of OCTA, making this endeavor well worth the effort.

## Figures and Tables

**Table 1:** Symbols or variables used and their meaning.

Symbol	Meaning
S	Complex OCT signal/field
*|*S*|*	Amplitude of the OCT signal
I = *|*S*|*^2^	Intensity of the OCT signal
∅	Phase of the OCT signal
S_m_	OCT field from one scatterer
SV	Speckle variance
cmOCT	Correlation mapping OCT signal
PV	Phase variance
Δ∅	Phase difference
ΔS	Complex field difference
CDV	Complex differential variance
R	Autocorrelation function
P	Power spectral density

**Table 2:** Classification of OCTA algorithms.

Category	Classification
OCT signal	Field vs. Intensity/Amplitude vs. Phase
Calculation	Variance/Difference vs. Correlation
Averaging method	Temporal vs. Spatial vs. Spectral
Normalization	Normalized vs. Non-normalized
